# Perforated Meckel's Diverticulum in a 22‐Year‐Old Male: A Case Report

**DOI:** 10.1002/ccr3.73231

**Published:** 2026-08-06

**Authors:** Munira Abdul Aziz, Hamza Shakil, Haris Mahmood, Rawdah Shakil, Muhammad Ali, Kamil Ahmad Kamil

**Affiliations:** ^1^ Department of Surgery Dow University Hospital Karachi Pakistan; ^2^ Dow International Medical College, Dow University of Health Sciences Karachi Pakistan; ^3^ Nasrat Bashir Curative Hospital Kandahar Afghanistan

**Keywords:** abdominal pain, diverticulectomy, Meckel diverticulum, perforation, peritonitis

## Abstract

Meckel's diverticulum (MD), the most common congenital anomaly of the gastrointestinal tract, results from incomplete regression of the vitelline duct. Although often asymptomatic, complications occur in 4%–7% of cases, with perforation being a rare but life‐threatening presentation that may mimic other acute abdominal emergencies. A 22‐year‐old Pakistani male was discovered with progressive abdominal pain, high‐grade fever, vomiting, and diarrhea. Clinical findings and radiographic evidence of pneumoperitoneum suggested enteric perforation. An exploratory laparotomy incidentally revealed a perforated MD located 60 cm proximal to the ileocecal junction. Diverticulectomy with limited small bowel resection was performed, and histopathology confirmed a true diverticulum without ectopic mucosa. The postoperative course was uneventful. This case draws attention to the diagnostic challenge of MD perforation, which may masquerade as other surgical emergencies. Early recognition and prompt surgical intervention are crucial for preventing morbidity, underscoring the need to consider perforated MD in young adults presenting with an acute abdomen.

## Key Clinical Message

Although rare, clinicians must acknowledge the differential diagnosis of a perforated Meckel's diverticulum in an adult presenting with an acute abdomen. Early diagnostic recognition and prompt surgical management are vital to prevent premature morbidity and mortality. Our case adds to the limited literature on adult MD‐related presentations and complications.

**Abbreviation**
MD: Meckel's diverticulum

## 1 Introduction

MD arises due to the incomplete regression of the embryonic vitelline duct. It represents the most frequently encountered congenital anomaly of the gastrointestinal tract in approximately 2% of the general population [1]. Meckel's diverticula are classified as true diverticula, as their walls include all the normal layers of the intestinal wall. While their exact location may vary, most are found in the ileum, typically within 100 cm of the ileocecal valve. Around 60% of Meckel's diverticula contain heterotopic mucosa, the majority of which consists of gastric tissue [2]. MD is typically asymptomatic and often identified incidentally during surgery or imaging studies, though it becomes clinically apparent through complications in approximately 4%–7% of cases [1]. Among the symptomatic cases, gastrointestinal bleeding is the most common presentation, followed by intestinal obstruction, diverticulitis, intussusception, volvulus, perforation, and, less frequently, neoplasms [2]. This case highlights a rare but serious complication of MD (diverticular perforation) in a rarely diagnosed and symptomatic age group (22‐year‐old adult) [1].

## 2 Case History and Examination

A 22‐year‐old Pakistani male with no known comorbidities, genetic conditions, or relevant family history presented to the emergency department with a 1‐week history of abdominal pain, fever, vomiting, and diarrhea. He reported being in his usual state of health until 1 week prior, when he developed a high‐grade fever (103°F), intermittent in nature and associated with chills and rigors, which was relieved by over‐the‐counter medications. The diarrhea was characterized by > 10 episodes per day of watery, non‐bloody stools. He also reported 2–3 episodes of non‐projectile vomiting. On general physical examination, the patient appeared toxic, acutely ill, and exhibited nasal flaring. Vital signs showed tachycardia at a heart rate of 132 beats per minute, tachypnea at 22 breaths/min, normoxemia at oxygen saturation of 98% on room air, and elevated blood pressure of 145/80 mmHg. Abdominal exam showed distention with board‐like rigidity. A digital rectal examination was deferred due to the presence of a rectal tube.

## 3 Methods

**FIGURE 1:**
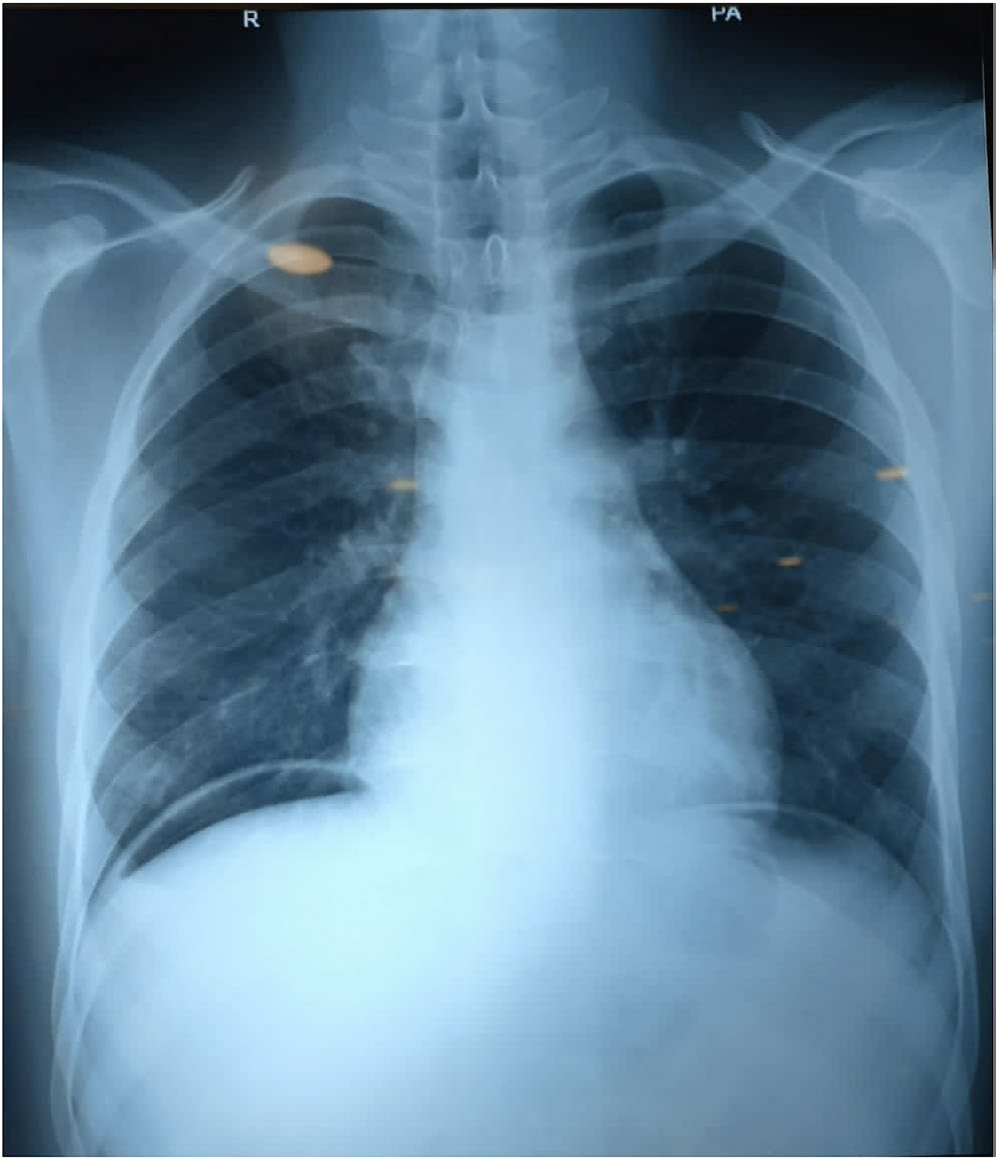
Chest X‐ray (anteroposterior view) demonstrating free air under the diaphragm.

**FIGURE 2:**
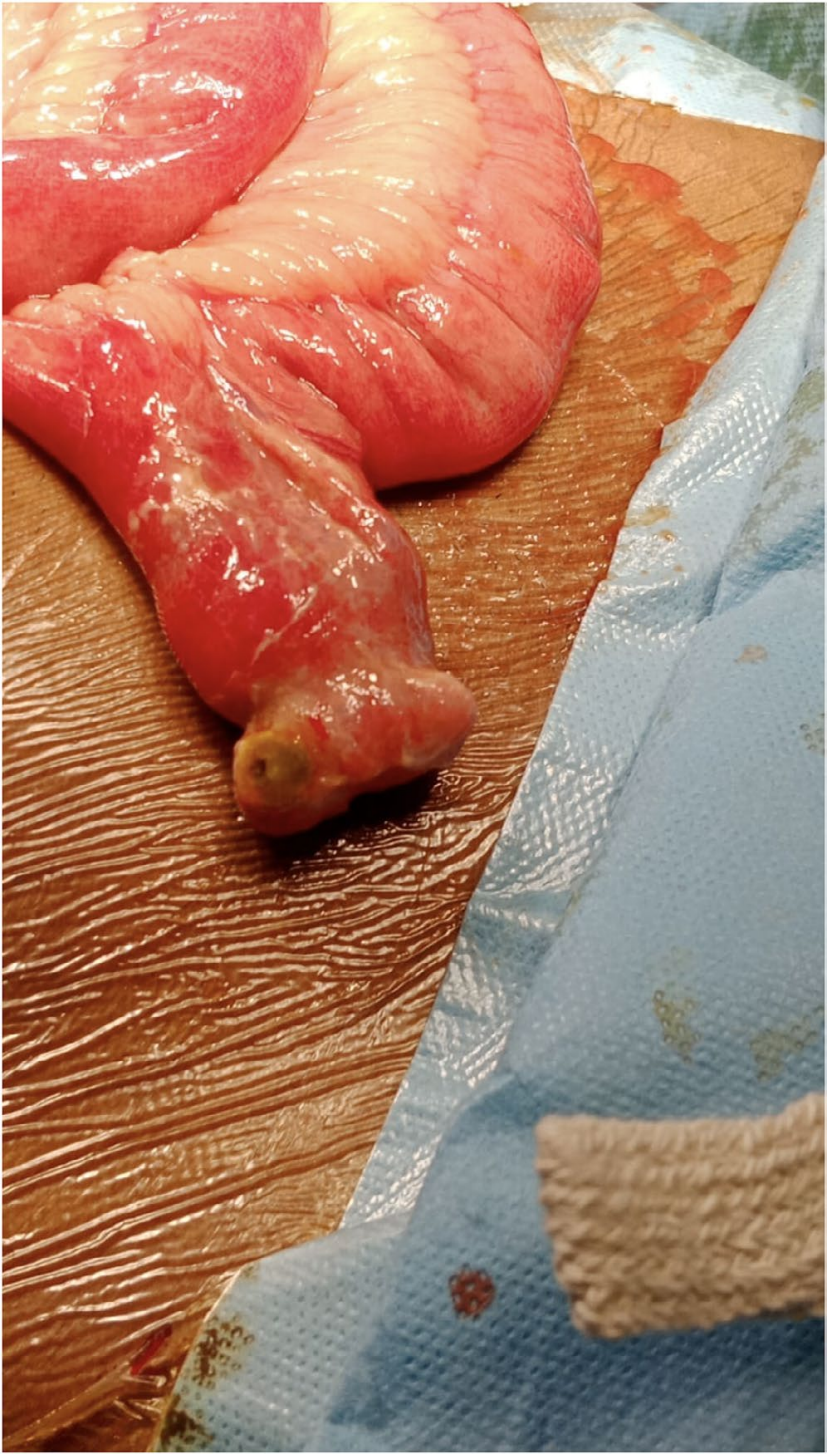
Inflamed, broad‐based diverticulum with apical perforation.

Moreover, a chest X‐ray (anteroposterior view) demonstrated free air under the diaphragm (Figure 1). Based on the clinical presentation, a working diagnosis of peritonitis secondary to enteric perforation was established. The patient was promptly started on intravenous fluids for resuscitation along with empiric broad‐spectrum antibiotics. Given the clinical presentation, operative intervention was deemed necessary. After gaining consent, the patient was taken to the operating room, where a diagnostic laparoscopy was conducted under general anesthesia. During the surgery, an inflamed bowel loop with surrounding turbid intraperitoneal fluid was observed along with evidence of inter‐bowel adhesions. Due to the presence of intra‐abdominal contamination with uncertain etiology, the procedure was converted to a midline exploratory laparotomy for further evaluation. A systematic examination of the small bowel was performed, which revealed a perforated MD approximately 60 cm proximal to the ileocecal junction. The diverticulum was inflamed, broad‐based, and perforated at its apex (Figure 2). Peritoneal washout was subsequently performed to address the intra‐abdominal contamination. A Meckel's diverticulectomy was performed in conjunction with a limited segmental small bowel resection. Approximately five centimeters of ileum proximal and distal to the diverticulum were resected. The affected bowel segment and diverticulum were resected using an 80‐mm linear cutter GIA stapler with a blue reload cartridge. Bowel continuity was restored via a side‐to‐side stapled anastomosis.

Histopathology confirmed a diverticulum consisting of all layers of the small intestinal wall, composed of intact villous‐glandular mucosa. However, no heterotopic mucosa was observed in our specimen.

## 4 Conclusions and Results

Postoperatively, the patient made an uneventful recovery. No postoperative complications were observed according to the Clavien‐Dindo classification. Subsequent clinical follow‐up revealed no adverse effects or complications (Table 1).

**TABLE 1:** Timeline of events.

Date	Event
Day −7	Onset of abdominal pain, fever, diarrhea, vomiting
Day 0	Emergency presentation
Day 0	Radiography showed pneumoperitoneum
Day 0	Diagnostic laparoscopy, converted to laparotomy
Day 0	Diverticulectomy with limited small bowel resection performed
Post‐operation	Uneventful recovery; discharged after follow‐up

## 5 Discussion

MD is the most prevalent congenital anomaly of the gastrointestinal tract, resulting from incomplete obliteration of the vitelline duct. It is found in approximately 2% of the population and is typically located on the antimesenteric border of the ileum, within 100 cm of the ileocecal valve. Although most cases remain asymptomatic, complications arise in 4%–7% of patients, which may include gastrointestinal bleeding (the most common presentation), followed by intestinal obstruction, diverticulitis, intussusception, perforation, and rarely neoplasms [1, 2].

Embryologically, MD comprises three main components: the omphalomesenteric duct, two vitelline arteries, and a single vitelline vein. Typically, the left vitelline artery regresses, while the right persists and develops into the superior mesenteric artery, which serves as the primary blood supply to the diverticulum. More specifically, MD is supplied by a terminal branch of the superior mesenteric artery, which crosses the ileum to reach the diverticulum [3, 4]. Progressive inflammation may compromise blood supply to the diverticulum, resulting in ischemia and eventual perforation, as was observed in our patient who presented with secondary peritonitis [5]. Other causes of perforation include obstruction of the diverticular lumen by a fecalith, which can trigger localized inflammation and tissue necrosis, ultimately leading to perforation. In rarer instances, perforation may result from the direct penetration of a foreign body [1].

In our case, the patient presented with an acute abdomen and signs of peritonitis, prompting radiographic evaluation that revealed pneumoperitoneum. Due to its non‐specific clinical presentation and wide range of differential diagnoses, establishing a preoperative diagnosis of MD can be difficult in both pediatric and adult patients. Although radionuclide imaging (Meckel's scan) is considered a useful tool for detecting MD, its effectiveness is significantly higher in children than in adults. In pediatric patients, the scan boasts an accuracy exceeding 90%, while in adults, its sensitivity drops to around 46%, making preoperative diagnosis challenging. As a result, fewer than 10% of adult cases are identified before surgery [6]. In our case, the 22‐year‐old patient was initially presumed to have an enteric perforation based on clinical presentation. However, during surgical exploration, a perforated MD was unexpectedly identified. This highlights the importance of considering MD perforation in patients with an acute abdomen.

Once symptomatic MD is identified, surgical management remains the standard of care. Treatment options include diverticulectomy, wedge resection, or segmental resection of the affected bowel segment. These procedures may be performed via an open or laparoscopic approach, depending on the surgeon's expertise and preference. Pediatric surgical literature indicates that both methods yield comparable outcomes in terms of hospital stay, postoperative complications, reoperation rates, and readmissions [7].

In most cases, Meckel's diverticula remain asymptomatic and are often discovered incidentally by surgeons during procedures performed for other, unrelated conditions. The management of incidentally discovered, asymptomatic MD remains controversial. While some advocate for prophylactic resection to prevent future complications, others caution that the risks associated with unnecessary surgery, especially in adults, may outweigh the benefits. This highlights the importance of individualized surgical judgment. However, Robin et al. [8], following an extensive review of the literature, reported that the morbidity associated with resection of incidentally discovered MD is significantly lower compared to that of symptomatic cases requiring surgical intervention. The authors proposed a risk scoring system incorporating four factors: male sex, patients younger than 45 years, diverticula longer than 2 cm, and the presence of a fibrous band. They recommended surgical resection of MD in cases where the total risk score was 6 or higher [8].

MD can present with a wide range of clinical manifestations, from painless rectal bleeding to bowel obstruction, perforation, and peritonitis. In our case, the clinical presentation closely resembled enteric perforation, representing a diagnostic challenge. This reinforces the need for heightened clinical suspicion, particularly in patients with unexplained abdominal pain progressing to peritonitis, after more common causes have been ruled out. Surgeons and clinicians should maintain a broad differential, keeping MD perforation in mind, even when the presentation aligns more with other acute abdominal emergencies.

## Author Contributions

**Rawdah Shakil:** resources, writing – review and editing. **Hamza Shakil:** formal analysis, investigation, writing – review and editing. **Muhammad Ali:** supervision, writing – review and editing. **Haris Mahmood:** methodology, project administration, supervision, validation. **Kamil Ahmad Kamil:** writing – review and editing. **Munira Abdul Aziz:** conceptualization, data curation, investigation, writing – original draft.

## Funding

The authors have nothing to report.

## Ethics Statement

The authors have nothing to report.

## Consent

Written informed consent was obtained from the patient for publication of this case report and accompanying images in accordance with the journal's patient consent policy.

## Conflicts of Interest

The authors declare no conflicts of interest.

## Data Availability

Data sharing is not applicable to this article as no datasets were generated or analyzed during the current study.
